# Metformin inhibits stromal aromatase expression and tumor progression in a rodent model of postmenopausal breast cancer

**DOI:** 10.1186/s13058-018-0974-2

**Published:** 2018-06-14

**Authors:** Erin D. Giles, Sonali Jindal, Elizabeth A. Wellberg, Troy Schedin, Steven M. Anderson, Ann D. Thor, Dean P. Edwards, Paul S. MacLean, Pepper Schedin

**Affiliations:** 1 Department of Nutrition & Food Science, Texas A&M University, 373 Olsen Blvd; 2253 TAMU, College Station, TX 77843 USA; 2 Anschutz Health & Wellness Center, University of Colorado Anschutz Medical Campus, Aurora, CO 80045 USA; 3 Department of Medicine, Divisions of Endocrinology, Metabolism, and Diabetes, University of Colorado Anschutz Medical Campus, Aurora, CO 80045 USA; 4 Department of Medical Oncology, University of Colorado Anschutz Medical Campus, Aurora, CO 80045 USA; 5 Department of Pathology, University of Colorado Anschutz Medical Campus, Aurora, CO 80045 USA; 6 Department of Cell, Developmental and Cancer Biology, Oregon Health & Science University, 3181 S.W. Sam Jackson Park Rd, Mailing Code: L215, Portland, OR 97239 USA; 7 Knight Cancer Institute, Oregon Health & Science University, 1130 NW 22nd Ave #100, Portland, OR 97239 USA; 8 Departments of Molecular & Cellular Biology and Pathology Immunology, Baylor College of Medicine, Houston, TX 77030 USA

**Keywords:** Obesity, Macrophage, Metabolism, Adipose, Tumor microenvironment, Liver

## Abstract

**Background:**

Obesity and type II diabetes are linked to increased breast cancer risk in postmenopausal women. Patients treated with the antidiabetic drug metformin for diabetes or metabolic syndrome have reduced breast cancer risk, a greater pathologic complete response to neoadjuvant therapy, and improved breast cancer survival. We hypothesized that metformin may be especially effective when targeted to the menopausal transition, as this is a lifecycle window when weight gain and metabolic syndrome increase, and is also when the risk for obesity-related breast cancer increases.

**Methods:**

Here, we used an 1-methyl-1-nitrosourea (MNU)-induced mammary tumor rat model of estrogen receptor (ER)-positive postmenopausal breast cancer to evaluate the long-term effects of metformin administration on metabolic and tumor endpoints. In this model, ovariectomy (OVX) induces rapid weight gain, and an impaired whole-body response to excess calories contributes to increased tumor glucose uptake and increased tumor proliferation. Metformin treatment was initiated in tumor-bearing animals immediately prior to OVX and maintained for the duration of the study.

**Results:**

Metformin decreased the size of existing mammary tumors and inhibited new tumor formation without changing body weight or adiposity. Decreased lipid accumulation in the livers of metformin-treated animals supports the ability of metformin to improve overall metabolic health. We also found a decrease in the number of aromatase-positive, CD68-positive macrophages within the tumor microenvironment, suggesting that metformin targets the immune microenvironment in addition to improving whole-body metabolism.

**Conclusions:**

These findings suggest that peri-menopause/menopause represents a unique window of time during which metformin may be highly effective in women with established, or at high risk for developing, breast cancer.

## Background

Obesity and type II diabetes are associated with an increased risk of breast cancer and poorer clinical prognosis, particularly in postmenopausal women. Over the past several years, many studies have reported decreased breast cancer incidence and/or mortality in diabetics receiving the widely prescribed antidiabetic drug metformin relative to those receiving other diabetic drugs [1–3]. Furthermore, these studies have found a dose-response relationship whereby women receiving the highest metformin dose for the longest duration show the most benefit [4, 5]. Despite the routine use of this drug, the anticancer mechanisms of metformin are not well understood. Additionally, some studies have failed to show a beneficial effect of metformin as an antitumor agent [6, 7]. Thus, there is ongoing interest in identifying patient populations who may benefit from metformin treatment, and the mechanisms by which metformin decreases cancer risk and/or improves tumor outcomes.

As a diabetic agent, metformin stabilizes glucose flux and reduces insulin resistance. It is used worldwide because of its low toxicity profile and low cost. Metformin activates AMP-dependent kinase (AMPK) to stimulate glucose uptake and glycogen synthesis, while suppressing gluconeogenesis, thereby improving whole-body insulin sensitivity. Beyond this, however, the exact mechanisms of action of metformin, even in diabetic patients, are not fully understood.

The antitumor activity of metformin has generally been attributed to its ability to decrease circulating insulin levels and improve whole-body metabolic health, as hyperinsulinemia has been associated with increased risk for breast cancer development, recurrence, and even death [8, 9]. There is also a growing body of literature to suggest that metformin may directly target both tumor cells and tumor stem cells [10–14]. These direct and indirect effects are not mutually exclusive, and it is possible that the effects of metformin involve a combination of the two.

Additional questions also remain regarding the patients and/or tumor subtypes for which metformin may be most effective. Preclinical studies suggest that at least some underlying whole-body metabolic dysfunction is needed to see beneficial effects of metformin, at least for breast cancer prevention [15, 16]. Clinical data to date are primarily derived from diabetic patients receiving metformin and, from the few clinical trials completed to date, it is unclear which patients benefit most [17–19]. The results of an ongoing clinical study are anticipated to shed light on this issue [20]. In-vitro studies have demonstrated potential therapeutic utility against all breast cancer cell lines tested, but there is emerging preclinical and clinical data suggesting that metformin may be more effective in treating specific tumor subtypes (reviewed in [21]). In cell lines, estrogen receptor (ER+) and tamoxifen-resistant breast cancers have been identified as a therapeutic target since metformin has been shown to inhibit the expression and function of ERα [12, 13]. Consistent with these in-vitro studies, a recent clinical study reported that metformin specifically benefits women with ER+ as well as human epidermal growth factor receptor 2 (HER2)-positive breast tumors [14]. Finally, we recently reported that tumor expression of organic cation transporter-2, which regulates metformin uptake, correlates with tumor responsiveness to metformin [22]. Thus, evidence suggests both host- and tumor-specific targets, and while understanding of the anti-cancer mechanisms of metformin are emerging, there are still many questions that remain unanswered. Here we focus on host biology, namely the metabolic dysregulation that occurs during the menopausal transition, as a potential window of increased metformin efficacy.

In a rodent model of ER+ postmenopausal breast cancer we have shown that, during ovariectomy (OVX)-induced weight gain, an impaired ability to clear excess nutrients from the circulation and store them in mammary adipose tissue correlated with tumor glucose uptake, markers of proliferation, and tumor progression [23]. Given these findings, we hypothesized that improving the whole-body metabolic response to excess calories during the window of weight gain that follows loss of ovarian function would decrease tumor growth and lead to improved tumor outcomes. We further hypothesized that both lean and obese animals would benefit from this treatment because they both experience overfeeding, rapid weight gain, and a decline in metabolic health in response to OVX [23, 24].

Here, we report that metformin effectively decreased mammary tumor burden in both obese and lean animals, and also prevented the formation of new tumors in the postmenopausal period. In addition to improving overall metabolic health, metformin also decreased the number of aromatase-positive, CD68-positive macrophages within the tumor microenvironment, suggesting a new role for metformin in targeting immune cells. We therefore propose that the menopause and the peri-menopausal window represent a unique opportunity for metformin therapy, specifically for women with existing ER^+^ tumors and/or those who may be at risk for the development of postmenopausal breast cancer.

## Methods

### Animal care and treatment

Female Wistar rats (100–125 g, 5 weeks of age) were purchased from Charles River Laboratories (Wilmington, MA) and housed at the University of Colorado Anschutz Medical Campus Center for Comparative Medicine (22–24 °C; 12-h/12-h light-dark cycle) with free access to water. All procedures were approved by the Institutional Animal Care and Use Committee.

### Animal model

Our OP-OR/OVX model of obesity and postmenopausal breast cancer was used as previously described [25]. We and others have shown that tumors that develop using this method are similar to human breast tumors with regard to: 1) the percentage of tumors that are intraductal; 2) the progression of histologic stages from hyperplasia, to carcinoma in situ, to invasive cancer; and 3) steroid receptor status [23, 25–27].

To induce obesity in these genetically susceptible rats, animals were individually housed in wire-bottomed metabolic cages to limit physical activity, and were given ad libitum access to a purified high-fat diet (HF; 46% kcal fat; Research Diets, New Brunswick, NJ; RD# D12344) for the duration of the study. Animals were ranked by their rate of weight gain from 5 to 15 weeks of age. Rats in the top and bottom tertiles of weight gain were matured to produce obese and lean animals, respectively. Rats from the middle tertile were removed from the study.

To induced mammary tumor formation, 55-day-old female rats (± 1 day) were given a single injection of the carcinogen 1-methyl-1-nitrosourea (MNU; 50 mg/kg; #ASI-701, Ash Stevens, Detroit, MI). Tumors were monitored by manual palpation at weekly intervals for the duration of the study and measured in three dimensions using digital calipers.

Body weight and food intake were monitored weekly, as previously described [24, 28]. Body composition was determined on the day of OVX, 5 weeks post-OVX, and again at the time of sacrifice by quantitative magnetic resonance (qMR; EchoMRI Whole Body Composition Analyzer; Echo Medical Systems, Houston, TX).

### Metformin treatment

In a rolling study design, animals were randomly assigned to either metformin treatment (2 mg/mL in the drinking water, *n* = 7 lean and *n* = 10 obese) or control group (water only, *n* = 8 lean and *n* = 9 obese) after at least one tumor in the animal reached a volume > 1 cm^3^. Rats were maintained on their respective treatments for the duration of the study. This dose was chosen based on results of pilot studies that produced plasma metformin levels similar to those used clinically, and because this dose demonstrated antitumor efficacy in obese rats in our previous short-term studies, with no observable negative side effects [22, 23]. Metformin treatment was initiated 1 week prior to OVX surgery to assure drug bioavailability during the critical window of rapid weight gain that immediately follows OVX. One week following the initiation of metformin treatment, the animals underwent surgical ovariectomy (OVX) under isoflurane anesthesia to mimic the postmenopausal state. At the time of OVX, biopsies of mammary tumors were obtained via fine needle aspiration (FNA).

### Plasma measurements

Tail vein blood was collected on the second diestrus day of the estrous cycle [24] during the week prior to OVX, at 5 weeks post-OVX, and again at the time of sacrifice. Blood was drawn during the latter part of the light cycle; plasma was isolated and stored at −80 °C until analyzed. Concentrations of insulin, leptin, amylin, and glucagon were simultaneously measured using the Rat Endocrine LINCOplex Kit 96 Well Plate Assay (RENDO-85 K; Millipore, St Charles, MO). Colorimetric assays were used to measure plasma free fatty acids (Wako Chemicals USA, Richmond, VA), glucose, triglycerides (TG), and total cholesterol (#TR15421, TR22321, and TR13521, respectively; Thermo Fisher Scientific, Waltham, MA).

### Histological staining and imaging

Sections of formalin-fixed paraffin-embedded tissue (4 μm) were stained with hematoxylin and eosin (H&E) using a Sakura autostainer (Sakura Finetek, Torrance, CA). Mammary tumors were classified histologically by the criteria of Young and Hallowes [29], and only adenocarcinomas were included in subsequent analyses. For immunohistochemical detection of progesterone (PR)-positive cells, 4 μm mammary tissue sections were stained with mouse monoclonal αPR, clone 6F11 at 1:100 (Vector Laboratories, Burlingame, CA). The dual localization of CD68 and aromatase was performed by staining for CD68 (Ab4059, Serotec, 1:200 dilution) sequentially followed by a mouse monoclonal aromatase antibody (clone 677) at a 1:100 dilution. The 677 monoclonal antibody was generated in one of the author’s laboratories (DPE) and has been validated extensively for specificity by immunohistochemistry (IHC) [30–33]. For CD68, mouse on rat secondary antibody (MRT621H, Biocare) for 30 mins was used followed by 3,3′-diaminobenzidine chromogen (DAB; K3467, Dako, Carpinteria, CA). The slides were sequentially stained with aromatase primary followed by Rat on mouse AP polymer (MALP521, Biocare) followed by Permanent red chromogen (K0640, Dako, Carpinteria, CA). The adipophilin primary antibody (LS-C348703, Lifespan Biosciences) was incubated on rat livers at 1:300 dilution for 60 min followed by mouse on rat secondary antibody (MRT621H, Biocare) for 30 min and DAB chromogen. Livers from lean and obese pre-OVX animals were obtained from a separate cohort of rats in which animals were terminated with the ovaries intact. All slides were counterstained with hematoxylin (S330130, Dako, Carpinteria, CA).

For mammary tumor ER and PR analysis, at least 11 tumors per group, and 8–10 fields/section (40× objective) were evaluated. Given the effectiveness of metformin in shrinking tumors in this study, the number of samples from metformin-treated rats with sufficient tissue to perform IHC analysis was limited; thus, for the CD68 and aromatase IHC in tumors and adjacent tumor border, our analysis was restricted to tumors from obese control and metformin-treated rats. CD68 and aromatase stained slides were scanned using an Aperio Scanscope3 system (Aperio, Vista, CA) at 20× magnification, corresponding to 0.43 μm per pixel which enables high-resolution access to the entire tissue section via a virtual image. Images were evaluated using Imagescope software and the signal captured and quantitated using Aperio algorithms. For tumor border analysis, the tumor boundary was outlined on H&E stained slides using the Aperio Annotation tool within Leica Image Scope (Leica Technologies, CA) by an MD pathologist (SJ). The tumor boundary images were then exported and overlaid (imported) onto an adjacent serial section dual stained for CD68 and aromatase using the Export/Import tool within Leica Image Scope. The tumor border was then defined as 100 μm external to the tumor boundary and captured using the Aperio Ruler tool. The Aperio system was also used for liver adipophilin quantitation, where a minimum of nine livers per group were evaluated.

### In-vitro macrophage differentiation

Rat macrophages were derived from a pooled bone marrow stock aspirated from femurs and tibias of 7-week-old female Wistar rats. Once isolated, marrow was cultured in vitro in Dulbecco’s Modified Eagle’s medium (DMEM) low glucose with 30% L929-cell conditioned media (as a source of macrophage colony-stimulating factor (M-CSF)) containing either 5 ng/mL lipopolysaccharide and 12 ng/mL interferon (IFN)-gamma to promote differentiation to an M1 phenotype, or 10 ng/mL interleukin (IL)-4 to promote an M2 phenotype. Following 48 h in differentiation medium, cells were rinsed twice with phosphate-buffered saline (PBS) and harvested in cell lysis buffer (50 mM Tris pH 7.4, 150 mM NaCl, 2.0 mM EDTA, 50 mM NaF, 5.0 mM sodium orthovanadate, 1% Triton X-100, 1% deoxycholate, 0.1% SDS) supplemented with Halt™ Protease and Phosphatase Inhibitor cocktail (Thermo Scientific). Lysates were centrifuged for 20 min at 14,000 g, and total protein concentration of the supernatant was determined by BioRad protein assay according to the manufacturer’s instructions.

### ProteinSimple© Western blotting and analysis

Protein levels of aromatase in M1 and M2 macrophages were measured using the Simple Western size-based capillary electrophoresis system (WES, ProteinSimple, San Jose, CA). Two different anti-aromatase primary antibodies were used, including Novus NB100-1596 (1:50 dilution) and clone #677 used for the previously described IHC (1:25 dilution). All procedures were performed according to the manufacturer’s protocol and immunodetection was conducted with default settings. Data were analyzed with ProteinSimple Compass software. Additional controls included the use of a blocking peptide against aromatase antibody #1 (Novus NB100-1596PEP) to verify the band size and specificity using this antibody on the WES system, and the use of forskolin-treated steroidogenic human granulosa-like tumor cells (KGN cells) to induced aromatase expression as a positive control with aromatase antibody #2.

### Statistical analysis

Data were examined with SPSS 24.0 software by ANOVA or χ^2^ analysis for nominal and ordinal data, respectively. Relationships between variables were assessed with the Spearman correlation coefficient. In some cases, data were analyzed by analysis of covariance with a specified covariate in the model.

## Results

### Baseline rat characteristics

#### Body weights and adiposity

**Fig. 1:**
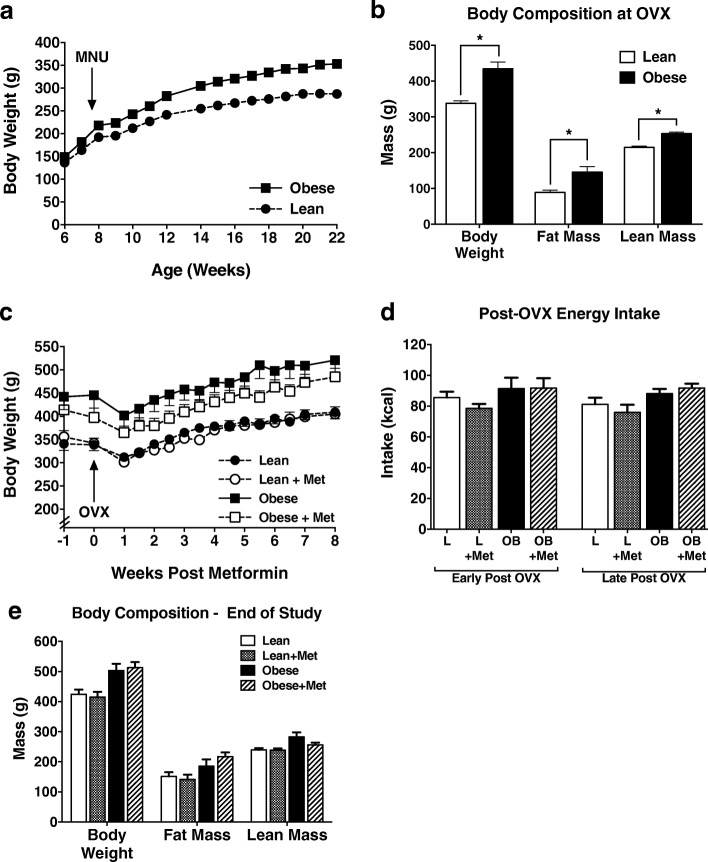
Body weight, composition, and food intake. **a** Body weights across the pre-ovariectomy (OVX) period for rats selected as lean or obese. **b** At the time of OVX (when animals had at least 1 tumor > 1cm^3^), obese (OB) rats were significantly heavier than the lean (L) group, and this was due to both higher fat and lean mass. **c** Body weights for lean and obese rats, with and without metformin (Met) treatment (2 mg/mL in drinking water), across the post-OVX period. Metformin was started at time −1 week; OVX occurred at week 0. Body weight was measured twice per week for the first 6 weeks following OVX, then weekly for weeks 7–8. A brief surgery-induced weight loss was followed by OVX-induced weight gain in both lean and obese animals, with no difference between metformin-treated and untreated animals. **d** Food intake is reported as the mean intake measured over the early (first 4 weeks) and late (weeks 5–8) post-OVX period. Metformin had no significant effect on food intake. **e** Body weight, fat mass, and lean mass measured by qMR at the end of the study. Obese rats had significantly higher body weight, fat mass, and lean mass than lean rats (*p* < 0.05), but metformin had no significant effect on these measures. *p < 0.05. MNU, 1-methyl-1-nitrosourea

Body weight and adiposity were measured pre-OVX, during OVX-induced weight gain, and bi-weekly until the study end. As we have shown previously [23–25, 34, 35], at the time of OVX surgery, mature obese rats had higher body weight (434 ± 19 g vs 338 ± 7 g) due to both higher lean mass (253 ± 5 g vs 215 ± 4 g) and percent body fat (31.9 ± 1.8% vs 25.9 ± 1.4%) when compared with their lean counterparts (Fig. 1a, b). In response to OVX, all animals, independent of pre-OVX obesogenic status, experienced a transient surgery-induced weight loss, followed by a significant amount of weight gain, as previously reported for female rats (Fig. 1c) [23–25]. Metformin did not significantly affect food intake during either the early (first 4 weeks) or late (weeks 5–8) post-OVX period (Fig. 1d); thus, it was not surprising that we found no differences in weight gain between the metformin-treated and control groups (Fig. 1c). Finally, at the study end, obese rats remained heavier than their lean counterparts, with higher fat and lean mass, with no statistical differences between the metformin-treated and control animals (Fig. 1e).

#### Plasmas measures

Blood samples were obtained at several time points throughout the study to measure both metabolic factors (1 week prior to OVX and again at the end of the study) and plasma levels of metformin (at the time of OVX, and 6 weeks post-OVX). Prior to OVX, obese animals had higher circulating TG than lean animals (Table 1; *p* < 0.05). By the study end, at 8 weeks post-OVX, we found no differences in plasma glucose, free fatty acids, or cholesterol between the lean and obese groups, likely because all animals were consuming a HF diet, gaining weight, and had developed metabolic disease. After 1 week of metformin treatment (at the time of OVX), plasma metformin averaged 1.15 ± 0.50 mg/mL, and this increased to 1.57 ± 0.47 mg/mL by 6 weeks. These levels are within the range reported in humans receiving daily metformin treatment [36, 37].

**Table 1:** Plasma characteristics prior to ovariectomy

	Lean	Obese
*n*	14	15
Glucose (mM)	5.74 ± 0.51	7.42 ± 0.51
Triglycerides (mM)	0.40 ± 0.05	0.78 ± 0.12^*^
Nonesterified fatty acids (μM)	536.8 ± 30.3	572.1 ± 42.0
Cholesterol (mM)	2.14 ± 0.09	2.33 ± 0.11

*p < 0.05

### Host responses

#### Metformin modestly improves markers of metabolic function

Despite achieving clinically relevant levels of metformin in the circulation, the impact of metformin on plasma metabolites was minimal in this study. One week of metformin treatment (the week prior to OVX) did not significantly alter glucose, insulin, leptin, glucagon, cholesterol, or nonesterified fatty acid (NEFA) levels (Table 2). A caveat of these metabolic analyses is that the animals were not fasted when plasma samples were taken, and this, combined with the ad libitum consumption of a diet high in fat, may have masked our ability to detect metformin-induced changes in levels of these metabolic markers. Metformin treatment tended to decrease two markers suggestive of improved metabolic health, namely TG (*p* = 0.058) and amylin (*p* = 0.095), which works synergistically with insulin to contribute to glycemic control (Table 2).

**Table 2:** Plasma characteristics at ovariectomy (after 1 week of metformin treatment)

	Control	Metformin	*p* value
Glucose (mM)	7.65 ± 0.82	6.91 ± 0.50	
Insulin (mM)	5.74 ± 2.59	3.31 ± 2.11	
Leptin (mM)	4.22 ± 1.82	5.32 ± 2.26	
Glucagon (mM)	3.71 ± 0.91	2.03 ± 0.54	
Cholesterol (mM)	1.81 ± 0.09	1.73 ± 0.09	
Nonesterified fatty acids (μM)	650 ± 64	658 ± 49	
Triglycerides (mM)	0.79 ± 0.14	0.51 ± 0.05**	0.058
Amylin (mM)	1.80 ± 0.69	0.65 ± 0.19**	0.095

**p < 0.10

**Fig. 2:**
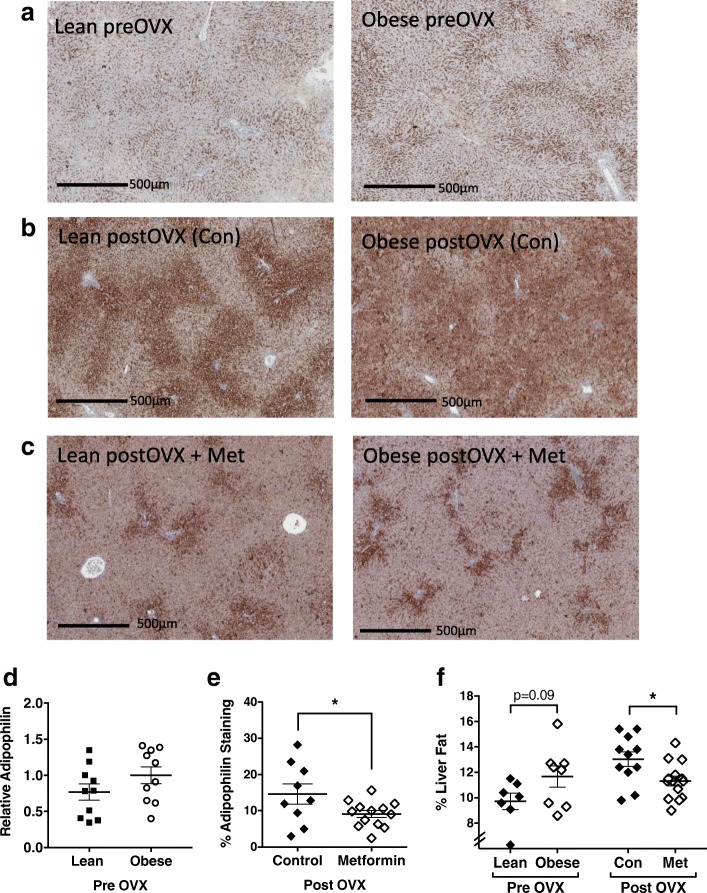
Hepatic lipid accumulation is increased with ovariectomy (OVX) and reduced with metformin (Met). Representative images are shown for adipophilin staining in livers from lean and obese rats **a** prior to OVX, **b** after OVX-induced weight gain, and **c** after OVX-induced weight gain with concurrent metformin treatment. Scale bars = 500 μm. **d** In pre-OVX rats, adipophilin levels are not significantly different between lean and obese rats. **e** Metformin treatment significantly decreased hepatic adipophilin staining when compared with controls. **f** qMR analysis of the percent fat in livers shows that absolute lipid content was not different between lean and obese rats pre-OVX, but post-OVX hepatic lipid accumulation was significantly decreased in metformin-treated rats compared with controls (Con). *p < 0.05

Since accumulation of lipid in the liver reflects an overall decline in metabolic health, we assessed the impact of metformin treatment on hepatic lipid accumulation. To quantify hepatic lipid accumulation, two independent measures were used: 1) semi-quantitative IHC staining for adipophilin, a marker of lipid droplet accumulation; and 2) qMR analysis of the liver, which measures the gross lipid content of the tissue. Figure 2 shows representative images of adipophilin-stained livers from lean and obese rats, prior to OVX and following 8 weeks of OVX-induced weight gain with or without metformin treatment. Prior to OVX, both lean and obese rats have low to moderate hepatic adipophilin staining, data consistent with overall normal or healthy livers (Fig. 2a). However, adipophilin is significantly increased in the post-OVX livers, regardless of pre-OVX adiposity (Fig. 2b). Quantitative assessment of these IHC-based data support minimal differences in hepatic lipid accumulation between lean and obese rats prior to OVX (Fig. 2d), followed by an extensive increase in lipid deposition in the livers of all animals following OVX. These observations highlight the role OVX plays in metabolic dysregulation independent of pre-existing adiposity status. In support of improved metabolic health in metformin-treated animals, adipophilin staining decreased significantly with metformin treatment (Fig. 2c, e; *p* < 0.05). qMR analysis further supports this finding as the percent fat in the liver decreased by 21% in metformin-treated animals compared to untreated controls (Fig. 2f). Together, these data show that OVX is associated with weight gain and hepatic lipid accumulation reflective of impaired metabolic health irrespective of pre-OVX obesity status. Treatment with metformin improves this marker of metabolic health when administered continuously across the menopausal (post-OVX) period.

#### Metformin decreases adipose tissue inflammation in post-OVX animals

The link between whole-body metabolic dysfunction and adipose tissue inflammation has been well established [38, 39], and work from our laboratory and others has suggested that inflammation induced by weight gain plays a role in promoting tumor growth [25, 40]. Thus, based on our finding that metformin decreased hepatic lipid accumulation, data consistent with improved whole-body metabolic health, we hypothesized that metformin may similarly alter mammary adipose tissue inflammation.

**Fig. 3:**
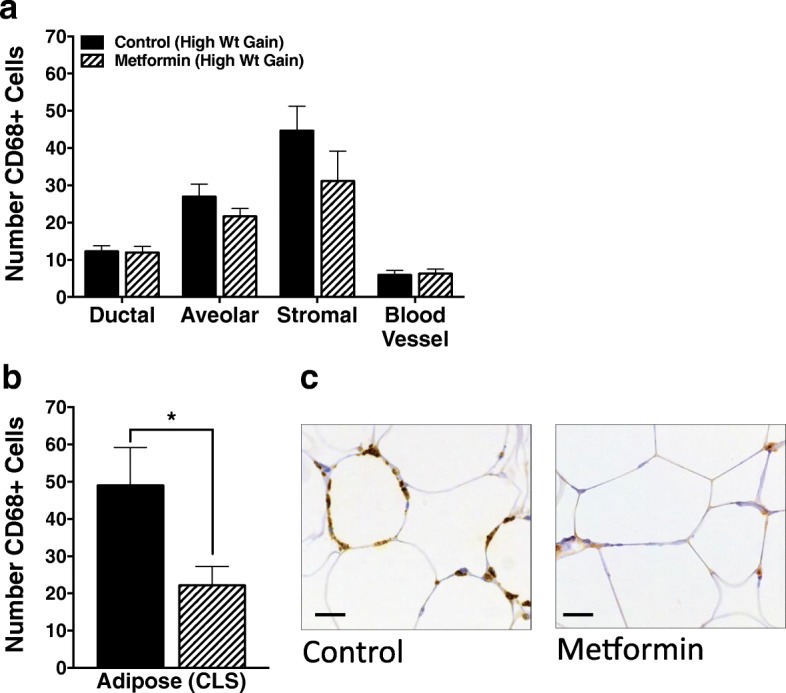
Metformin decreases the number of adipose-associated mammary macrophages. Quantification and representative images of CD68^+^ macrophages in normal mammary adipose tissue from metformin-treated and control rats experiencing high weight (Wt) gain in the post-OVX period. Rate of weight gain during the first 4 weeks after OVX was measured and those above the median were classified as high weight gainers and those below the median as low weight gainers. Mammary adipose was stained for CD68 as a macrophage marker. **a** Metformin did not affect the number of ductal, alveolar, stromal, or blood vessel-associated CD68^+^ cells, but **b** metformin decreased the number of CD68^+^ cells associated with adipocytes and crown-like structures (CLS) in rats rapidly gaining weight following OVX. **c** Representative images from control and metformin-treated adipose tissue are shown. Scale bars = 20 μm

Crown-like structures, comprised of macrophages in close proximity to adipocytes, are a hallmark of chronic adipose tissue inflammation, and their presence in the mammary gland or breast has been associated with insulin resistance in both rodent and human studies [41]. In the breast, the macrophage marker CD68 has been used to identify crown-like structures that may otherwise be missed using H&E staining [42]. Thus, we quantified the number of CD68-positive (CD68^+^) cells in IHC-stained sections of mammary tissue from nontumor bearing mammary glands taken at the study end. We stratified animals into those with high versus low rates of weight gain during the post-OVX period, which are predicted to have high versus low inflammatory milieus, respectively. While we found no overall differences in the number of CD68^+^ macrophages associated with mammary ducts, alveoli, stroma, or blood vessels (Fig. 3a), metformin treatment in the high-weight-gain animals significantly decreased the number of adipose-associated macrophages forming crown-like structures (CLS) (Fig. 3b, c). Furthermore, in animals with low post-OVX weight gain, metformin had no effect on the number of adipose-associated CD68^+^ cells (36.8 ± 10.8 vs 25.7 ± 4.8, metformin vs control; *p* = 0.321), suggesting that metformin may be specifically effective at decreasing the inflammation induced by weight gain following OVX. The decrease in crown-like structures in metformin-treated high-weight-gain animals suggests that, in addition to improving whole-body metabolic function (liver data above), metformin also contributes to adipose-specific metabolic improvements. Combined, these studies support beneficial effects of metformin on both whole-body metabolic health and mammary adipose tissue inflammation, which we anticipated may underlie the antitumor effects of metformin.

### Tumor response to metformin

Epidemiological data have, for the most part, focused on the beneficial role of metformin in improving tumor outcomes in individuals with type 2 diabetes, who are also commonly overweight and/or obese [43]. However, based on the knowledge that rapid weight gain occurs following OVX, regardless of adiposity phenotype, as well as the beneficial effects of metformin after OVX described above, we hypothesized that metformin would be effective in animals when administered during the period of OVX-induced weight gain, regardless of their pre-OVX obesogenic status.

As previously reported in this rodent model, lean and obese animals did not differ in the number of tumors prior to OVX (1.89 ± 0.34 vs 1.90 ± 0.42, lean vs obese), consistent with epidemiologic data showing minimal impact of obesity on breast cancer risk in premenopausal women [44]. Also, we found that pre-OVX adiposity (lean vs obese) did not affect post-OVX tumor response to metformin, with both groups experiencing similar OVX-induced weight gain and reduction in tumor burden with metformin treatment. Thus, for tumor burden and for subsequent analyses, data from lean and obese groups were combined and data are presented as control versus metformin-treated, unless indicated otherwise.

**Fig. 4:**
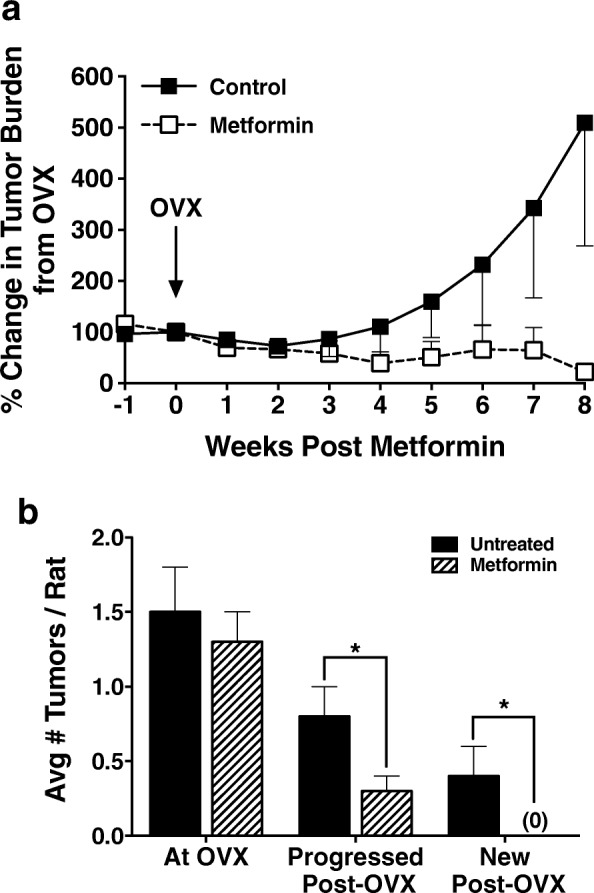
Metformin suppresses mammary tumors in a rat model of postmenopausal breast cancer. **a** Metformin treatment decreases tumor growth during the 8-week, postovariectomy (OVX) follow-up period. **b** Eight weeks of metformin treatment resulted in fewer tumors that progressed, no new tumors, and a decrease in overall tumor progression. Data from lean and obese groups are combined since differences between groups were not detected. *p < 0.05

In the first 2 weeks following OVX, tumor burden decreased slightly in all animals regardless of metformin treatment, as predicted, based on the estrogen dependence of MNU-induced tumors. Beginning at 3 weeks post-OVX, in untreated rats, tumor growth increased significantly and continued to increase in size for the duration of the 8-week study (Fig. 4a). Conversely, treatment with metformin prevented this post-OVX tumor growth and also resulted in tumor regression such that many tumors present at the time of OVX were no longer palpable at 8 weeks post-OVX (Fig. 4a). Specifically, mean tumor burden in the metformin-treated rats was 86% lower than in their untreated counterparts. This decrease in tumor burden is primarily due to the fact that metformin prevented tumor progression after OVX, rather than directly causing tumor regression per se. At the study end, overall, metformin-treated animals had significantly fewer tumors per rat that progressed (0.8 ± 0.3 vs 0.3 ± 0.1), no new tumors that emerged (0.4 ± 0.2 vs 0), and fewer tumors remaining (1.7 ± 0.4 vs 0.8 ± 0.1), irrespective of pre-OVX obesogenic status (Fig. 4b).

### Metformin-induced changes in the tumor microenvironment

To explore potential mechanism(s) by which metformin exerted its antitumor effects, we returned to the demonstrable links between obesity, local estrogen production, and ER^+^ breast cancers. In the postmenopausal setting, aromatase is the key enzyme responsible for estrogen production, as it converts testosterones to estrogens. Importantly, increased expression of aromatase in stromal adipocytes and associated vascular cells has been linked to inflamed adipose tissue in obese and overweight rodents and women [42, 45], and is thought to be the local source of growth promotion for breast cancers in the postmenopausal setting. Thus, one potential mechanism of action proposed for the anti-tumor effects of metformin in our postmenopausal breast cancer model is through the inhibition of stromal-derived aromatase.

#### Metformin decreases aromatase-positive, CD68^+^ macrophages in the tumor border

**Fig. 5:**
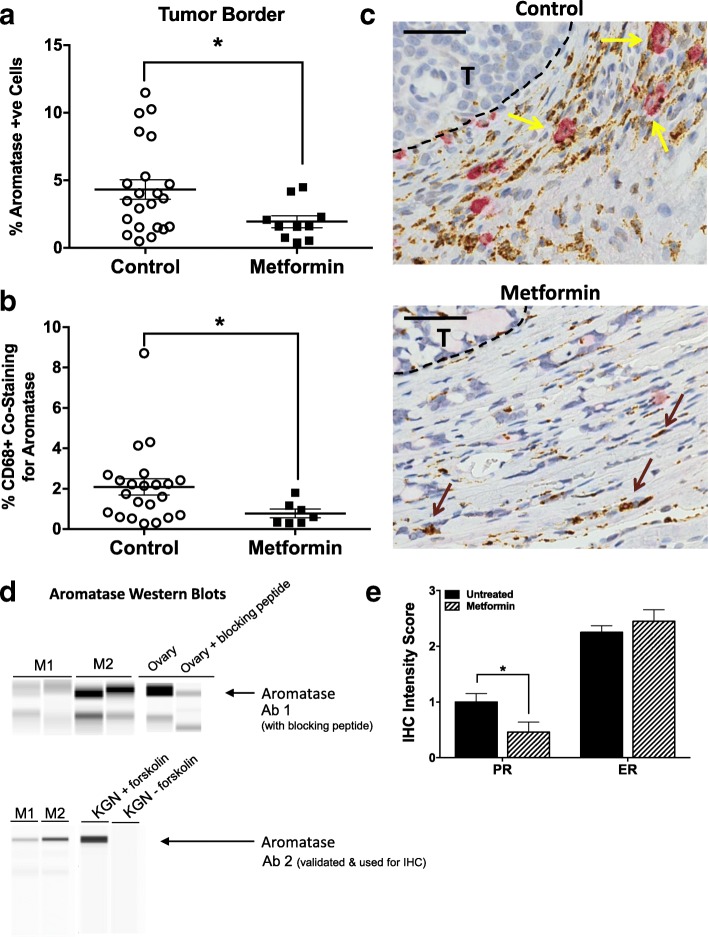
Metformin decreases aromatase-positive, tumor-associated macrophages. **a** Quantification of aromatase-positive cells and **b** CD68-positive (CD68^+^) macrophages costaining for aromatase in the tumor border of tissues from control or metformin-treated rats. **c** Representative control and metformin-treated IHC images of CD68 and aromatase dual staining in tissue bordering mammary tumors (T) (arrows: yellow = CD68^+^aromatase^+^, brown = CD68^+^; scale bar = 50um). **d** Aromatase expression by Western blotting (WES system) from in vitro activated M1 and M2 rat macrophages using two different primary antibodies (Ab #1: Novus NB100-1596; Ab #2: clone 677, Baylor College of Medicine). Controls include ovary (positive control) and ovary in which the primary antibody was preincubated with an aromatase blocking peptide for 1 h, as well as KGN cells with (positive control) and without (negative control) forskolin treatment. **e** IHC intensity score for ER and PR (0 = no stain; 1 = weak; 2 = moderate; 3 = strong staining). *p < 0.05

To investigate if metformin exerted an antitumor effect by modulating aromatase expression in inflamed tissues, we returned to our observation that metformin reduced the number of CD68^+^ macrophages present within the mammary adipose compartment. While several studies have reported that aromatase expression/production occurs in mammary stromal vascular cells, there is only one other known report of CD68^+^ macrophages expressing aromatase [46]. Using a quantitative IHC approach with an IHC-validated antibody [30–33], we first evaluated whether metformin decreased aromatase levels in either mammary tumors or the surrounding tumor microenvironment. While metformin did not affect aromatase levels within the mammary tumor cells, we did find a significant decrease in the number of aromatase-positive stromal cells in the tumor border of metformin-treated animals compared with controls (Fig. 5a). Dual staining for aromatase and the macrophage marker CD68^+^ revealed the aromatase-positive stromal cells to be heterogeneous, comprising CD68^+^ macrophages and additional unidentified stromal cell population(s). Furthermore, metformin specifically decreased the number of CD68^+^, aromatase-positive macrophages (Fig. 5b, c). This effect was specific to the tumor border since differences within the tumors themselves were not detected. This confirms the work of Mor and colleagues [46] identifying CD68^+^ macrophages as a cell type responsible for aromatase production, and the first report that macrophage expression of aromatase is metformin responsive.

#### Metformin targets a subset of M2-like macrophages

Because macrophages exhibit a wide range of antitumor abilities, which are determined in part by their polarization state, we next assessed if aromatase expression is influenced by macrophage phenotype. M1, or proinflammatory macrophages, are involved in antigen presentation, immune surveillance and killing of cells with foreign antigens, including tumor cells, and thus are considered tumor suppressive [47–49]. M2, or alternatively activated macrophages, represent the other end of the polarization spectrum, and are considered immunosuppressive and can promote tumor progression [50–52]. Using in vitro activated rat M1 and M2 macrophages, we found higher levels of aromatase protein in M2 compared to M1 polarized rat macrophages, as assessed by Western blot (Fig. 5c). Data were confirmed using three distinct sources of M1 and M2 polarized rat macrophages, and two different aromatase antibodies. Antibody specificity was also verified using a commercially available aromatase blocking peptide (Fig. 5d). Similar results have been obtained using murine macrophages (data not shown). In summary, we interpret these data to suggest that a specific subtype of aromatase-positive M2 polarized macrophages are elevated with OVX-induced weight gain, associate with increased tumor burden in our postmenopausal breast cancer rat model, and are suppressed by metformin.

#### Metformin decreases local estrogen levels and reduces ER signaling in tumors

One prediction of reduced macrophage-derived aromatase expression within the tumor-bearing mammary glands would be decreased ER signaling in the tumors. As a read out of ER signaling, we measured expression of the well-established ER response gene, the progesterone receptor (PR), by IHC. As expected, PR expression was decreased in metformin-treated tumors relative to controls (Fig. 5e). This observation is consistent with metformin acting, in part, by reducing ligand-dependent estrogen signaling within the mammary tumor microenvironment. Importantly, we found no difference in ER levels (Fig. 5e), likely because ER levels are high in these tumors after OVX. Thus, our data suggest that through actions on mammary macrophages, metformin decreases local aromatase levels, leading to lower levels of estrogens and decreased ER activation within the tumors, which could ultimately decrease tumor growth.

## Discussion

Menopause represents a lifecycle window of breast cancer risk that may be highly amenable to interventions that decrease risk. During menopause, energy balance, circulating hormones, chemokines and cytokines, and body fat distribution are in flux, and this is also the critical time when the tumor-promoting effects of obesity emerge [53–55]. Thus, interventions targeting the metabolic flux of menopause may effectively reduce breast cancer incidence and/or lethality. Using a rat model of postmenopausal breast cancer, our goal was to determine if targeting metformin treatment to the window of ‘menopause’-induced weight gain could decrease tumor growth and improve tumor outcomes. Similar to menopause in women, OVX in this model induced weight gain and increased adiposity in all animals, regardless of their lean/obese status prior to OVX. Weight gain was associated with a decline in metabolic health, as demonstrated by increased liver fat deposition and adipose-tissue inflammation. Specifically, we identified a subtype of aromatase-positive, M2-like macrophages to be elevated in mammary adipose tissue post-OVX. Within this context of OVX-induced metabolic dysfunction, metformin decreased the size of existing tumors and prevented formation of new tumors. The antitumor effects of metformin were associated with a decrease in adipose inflammation, measured by a reduction in the number of aromatase expressing CD68^+^ macrophages. Overall, our data suggest that a subtype of aromatase-positive, M2-like macrophages are elevated with OVX-induced weight gain, providing a growth advantage to ER^+^ tumors in the absence of ovarian hormones. These macrophages are targeted by treatment with metformin, possibly mitigating the protumorigenic effects of OVX-induced weight gain through estrogen deprivation. While nonaromatase-dependent mechanisms of metformin most certainly also contribute to tumor reduction in this model, this newly identified mechanism of action warrants further investigation.

This study further supports our ‘dual requirement’ hypothesis of obesity and postmenopausal breast cancer. Our early work in this model established that both impaired metabolic regulation that underlies obesity and a positive energy imbalance are required for the emergence of obesity-associated tumor promotion after menopause. This combination of impaired metabolism and postmenopausal weight gain has direct effects on mammary tumors, specifically increasing tumor expression of PR, promoting a glycolytic/lipogenic gene expression profile, and promoting tumor glucose uptake [23]. More recently, we have also shown that this combination of obesity and OVX-induced overfeeding leads to nuclear localization of the androgen receptor, which promotes the growth of ER+ tumors under conditions of low estrogen availability after OVX [35]. Importantly, in that study we found a role for the inflammatory cytokine IL-6 in sensitizing breast cancer cells to low testosterone levels. Our current study now extends this work to suggest that rapid weight gain following OVX/menopause is associated with increased aromatase expression in mammary macrophages. Work in endometrial cancer has demonstrated that tumor cell production of IL-6 leads to upregulation of aromatase in stromal cells, creating a cycle that drives tumor proliferation [56]. A similar IL-6-mediated increase in aromatase gene expression has been demonstrated in murine macrophages [57]. The existence of a similar paracrine mechanism in the context of postmenopausal breast cancer warrants further investigation.

The combination of impaired metabolic health and menopause-induced weight gain were likely critical to the anticancer effects of metformin observed in this study. A review of the literature would suggest that metformin is most effective when one or more of the following are present: 1) consumption of a moderate to high-fat diet; 2) poor metabolic health (insulin insensitivity, metabolic disease, etc.); 3) weight gain; and/or 4) increased adiposity (overweight or obesity). Our previous work demonstrating beneficial effects of metformin have all been conducted in animals consuming a high-fat diet [22, 23]. There are several examples where metformin had minimal or no impact on mammary tumor outcomes in the context of a low-fat diet [15, 58, 59]; however, in studies where medium [60] or high-fat [61] diets were used, metformin improved tumor outcome. Beneficial effects of metformin have also been reported in a study where 5% sucrose was added to the water of the animals [62], suggesting that this may have been sufficient to impair the metabolic health of these animals to an extent where the effects of metformin could be realized.

While the role of tumor-associated macrophages in breast cancer development and progression has been studied extensively over the past decade (reviewed in [63]), there are only a limited number of studies that have focused specifically on macrophage production of aromatase. Using IHC staining on serial sections, Mor and colleagues [46] demonstrated the presence of CD68^+^, aromatase-positive macrophages both around and within human breast cancers. Using in vitro assays, they extended this work to show that aromatase expression and activity is acquired by tissue-activated macrophages but not by their circulating monocyte precursors. Furthermore, conditioned medium from activated macrophages was sufficient to stimulate the growth of estrogen-responsive MCF-7 cells—an effect blocked by the aromatase inhibitor letrazol. This demonstrates that, at least in vitro, macrophages can produce sufficient levels of estrogens to stimulate the growth of estrogen-responsive breast cancer cells. Our findings are the first, to our knowledge, to identify aromatase expression as a feature of a subpopulation of protumorigenic M2-like mammary macrophages that arise in the context of obesity. We speculate that, in our in-vivo model, locally produced estrogens reach sufficient levels to activate ER in mammary tumors, and the ability for metformin to decrease this local production of estrogen contributes to its antitumor effects. The question whether inflammatory cytokines such as IL-6 cooperate with stromal-derived estrogen and sensitize breast cancer cells to ER, as we observe for testosterone signaling through the androgen receptor [35], remains to be determined.

Our data build upon the work of Dannenberg’s group who have demonstrated a causal link between obesity-induced inflammation and aromatase expression in the mammary gland. Their work shows that, in obese mice, release of free fatty acids from adipocytes activates NF-kB in the stromal vascular fraction of adipose tissue, which increases proinflammatory cytokine production [45]. In cell culture models, they have demonstrated that that these proinflammatory mediators (tumor necrosis factor (TNF)α, IL-1β, and prostaglandin E_2_(PGE_2_)) produced by cells in the stromal fraction of mammary glands from obese mice stimulate aromatase in preadipocytes [45]. They have further extended these findings to demonstrate increased inflammation, aromatase expression, and aromatase activity in the breast of overweight and obese women [42] and in a subset of nonoverweight women (body mass index (BMI) < 25 kg/m^2^) who had underlying systemic metabolic dysfunction [41]. Our work extends these pioneering studies and indicates that M2-like macrophages themselves can produce aromatase. In our rat model, mature mammary adipocytes do not appear aromatase-positive, and estradiol in rat mammary adipose tissue was below the level of detection by mass spectrometry [35]. It is possible that the role of adipocytes in aromatase production could be model- or context-dependent. However, combined, these data highlight the fact that many stromal cell populations may contribute to local aromatase production under different conditions.

## Conclusions

In conclusion, our work from preclinical models demonstrates that metformin specifically targets macrophage production of aromatase in the mammary adipose depot, identifying a potential novel antitumor mechanism of action for metformin. Additional studies are needed to confirm the relevance in women. Nonetheless, our findings provide the rationale for testing the efficacy of metformin in higher-risk populations, such as peri-menopausal or menopausal women with underlying metabolic disease. The menopause transition represents a lifecycle window of opportunity that may be specifically sensitive to the beneficial effects of metformin, and use of this agent during this time could improve outcomes for many women at risk for or those with established postmenopausal breast cancer.

## Abbreviations

AMPK: AMP-dependent kinase
BMI: Body mass index
CD68+: CD68 positive
CLS: Crown like structure
ER: Estrogen receptor
ER+: Estrogen receptor positive
FNA: Fine needle aspiration
H&E: Hematoxylin and eosin
HER2: Human epidermal growth factor receptor 2
HF: High fat
IHC: Immunohistochemistry
MNU: 1-methyl-1-nitrosourea
NEFA: Nonesterified fatty acid
OVX: Ovariectomy
PGE2: Prostaglandin E2
PR: Progesterone receptor
qMR: Quantitative magnetic resonance
TG: Triglycerides
TNFα: Tumor necrosis factor alpha
